# Identification of candidate genes or microRNAs associated with the lymph node metastasis of SCLC

**DOI:** 10.1186/s12935-018-0653-5

**Published:** 2018-10-19

**Authors:** Zhonghao Wang, Bei Lu, Lixin Sun, Xi Yan, Jinzhi Xu

**Affiliations:** Department of Thoracic Surgery, The Fourth Affiliated Hospital of Harbin Medical University, No. 37, Yiyuan Street, Nangang District, Harbin, 150001 Heilongjiang China

**Keywords:** Metastasis, Lymph node, Small cell lung cancer, Survival

## Abstract

**Background:**

Small cell lung cancer (SCLC) is a highly malignant cancer, and over 70% of patients with SCLC present with the metastatic disease. We aimed to explore some novel differentially expressed genes (DEGs) or microRNAs (miRNAs) associated with the lymph node metastasis of SCLC.

**Methods:**

The DEGs between the metastasis and cancer groups were identified, and GO functional and KEGG pathway enrichment analyses for these DEGs were implemented. Subsequently, the protein–protein interaction network and subnetwork of module were constructed. Then the regulatory networks based on miRNAs, transcription factors (TFs) and target DEGs were constructed. Ultimately, the survival analysis for DEGs was performed to obtain the DEGs related to the survival of SCLC.

**Results:**

Here, 186 upregulated (e.g., GSR, HCP5) and 144 downregulated DEGs (e.g., MET, GRM8, and DACH1) were identified between the SCLC patients with lymph node metastasis and without lymph node metastasis. GRM8 was attracted to the G-protein coupled receptor signaling pathway. Besides, miR-126 was identified in the miRNAs-TFs-target regulatory network. GRM8 and DACH1 were all regulated by miR-126. In particular, GSR and HCP5 were correlated with survival of SCLC patients.

**Conclusion:**

MiR-126, DACH1, GRM8, MET, GSR, and HCP5 were implicated in the lymph node metastasis process of SCLC.

## Background

Lung cancer (LC) is a malignant lung tumor characterized by unbounded cell growth in the lung tissues [1]. It is estimated that there are approximately 4,291,600 new cancer cases in China in 2015, and LC is still the main factor for cancer-associated death [2, 3]. Currently, LCs are frequently divided into small cell lung cancer (SCLC) and non-small cell lung cancer (NSCLC), and 10–15% of LCs are SCLC [4, 5]. SCLC, a poorly differentiated and aggressive type of LC, presents an early metastases, fleetly growth rate, and poor prognosis with a lower overall 5-year survival rate [6–8]. However, the molecular determinants of SCLC metastasis are unclear. Thus, it is essential to explore the determinants to prevent SCLC metastasis.

Lymph nodes, the central trafficking hubs for recirculating immune cells, are widely present throughout the body [9]. Conceivably, tumor cells could migrate into the lymph nodes and rapidly spread to other organs [10]. Activator protein-1 (AP-1), a transcription factor (TF), regulates the gene expression in response to various stimulus [11]. It has been reported that the overexpression of AP-1 is related to the lymphatic metastasis of LC [12]. Intriguingly, the abnormal expression of genes regulated by AP-1 was also involved in the process of lymphatic metastasis. For example, previous studies found that the overexpressions of urokinase type plasminogen activator (u-PA) and u-PA receptor (u-PAR) were correlated with the lymphatic metastasis of LC [13, 14]. In particular, the overexpression of AP-1 contributes to the overexpressions of u-PA and u-PAR. Recently, other studies demonstrated that cyclooxygenase-2 (COX-2) overexpression is well related to the lymphatic metastasis of LC [15, 16]. In the promoter regions of COX-2 genes, there is a binding site of AP-1 [17]. Furthermore, in metastatic lymph nodes, the vascular endothelial growth factor C (VEGF-C) overexpression is closely correlated with the lymph node metastasis of NSCLC [18]. These all findings revealed that the abnormal expression of TFs or genes were associated with the lymphatic metastasis of LC, especially for the NSCLC. However, yet little is known about the processes of tumor cell migration and lymph node metastasis in SCLC [19]. Therefore, we aimed to explore some novel differentially expressed genes (DEGs) related to the lymph node metastasis process of SCLC, and the potential mechanism would be elucidated.

The bioinformatics analysis methods were carried out for screening DEGs correlated with the lymph node metastasis process of SCLC. Firstly, the DEGs between the metastasis and cancer groups were screened. Afterwards, Gene Ontology (GO) functional and Kyoto Encyclopedia of Genes and Genomes (KEGG) pathway enrichment analyses for the DEGs were implemented to obtain the potential functions of DEGs. Afterwards, the protein–protein interaction (PPI) network and subnetwork of module were established. Then the regulatory networks based on microRNA (miRNAs), TFs and target DEGs were constructed. Ultimately, the survival analysis for DEGs was performed to obtain the DEGs related to the survival of SCLC.

## Materials and methods

### Microarray data

The gene expression profiling GSE40275 was obtained from the Gene Expression Omnibus (GEO) database (http://www.ncbi.nlm.nih.gov/geo/) [20], which included 4 SCLC samples with the lymph node metastasis (metastasis group, GSM990225, 226, 227, 247) and 6 SCLC samples without the lymph node metastasis (cancer group, GSM990214, 215, 216, 217, 218, 246). All samples were collected from the SCLC patients and detected through the GPL15974 Human Exon 1.0 ST Array [CDF: Brainarray Version 9.0.1, HsEx10stv2_Hs_REFSEQ] platform.

### Data preprocessing and DEGs screening

We downloaded the raw CEL data and used the Oligo package (ver.1.38.0) (http://bioconductor.org/help/search/index.html?q=oligo/) [21] in R language to pre-process all the data by performing background correction, conversion of original data and quartile data normalization. In order to remove the probes that cannot match the gene symbol, probes were annotated by the annotations file. The average value of different probes would serve as the final expression level of gene if different probes were mapped to the same gene symbol. DEGs were screened via the classical Bayesian method provided by limma package (ver. 3.30.13, http://www.bioconductor.org/packages/2.9/bioc/html/limma.html) [22]. The setting of thresholds was p value < 0.05 and |log fold change (FC)| ≥ 1.5.

### Functional and pathways enrichment analyses

GO [23] and KEGG pathway [24] analyses for DEGs were implemented utilizing the Database for Annotation, Visualization and Integration Discovery (DAVID) (ver. 6.8, https://david-d.ncifcrf.gov/) [25] tool. The number of enrichment genes (count number) ≥ 2 and p value < 0.05 were regarded as the thresholds criteria.

### PPI network and subnetwork of module analyses

The Search Tool for the Retrieval of Interacting Genes (STRING) (ver. 10.5, http://www.string-db.org/) [26] database was carried out to analyze the protein–protein interactions of DEGs. The DEGs acted as the input gene set, while the homo sapiens served as species. The PPI score was set as 0.4. Thereafter, the Cytoscape (ver. 3.6.0, http://www.cytoscape.org/) software was applied to construct the PPI network. The significant clustering modules were analyzed using Molecular Complex Detection (MCODE) (ver. 1.5.1, http://apps.cytoscape.org/apps/MCODE) [27] plugin. The threshold value was set as score ≥ 5.

### MiRNAs-TFs-target regulatory network analyses

The iRegulon (ver. 1.3, http://apps.cytoscape.org/apps/iRegulon) [28] plugin in Cytoscape was performed to predict and analyze the interaction pairs of TF-target gene in the PPI network. The parameters were set as follows: the minimum identity value among orthologous genes was set as 0.05, and the maximum false discovery rate on motif similarity was set as 0.001. The higher score of Normalized Enrichment Score (NES) in output results presented the more reliable results. The TF-target interaction pairs whose NES > 4 were selected for further study. Afterwards, the miRNAs-target were predicted on the basis of WebGestal (http://www.webgestalt.org/option.php) using the Overrepresentation Enrichment Analysis (ORA) method. The setting of threshold was count number ≥ 2 and p value < 0.05. Ultimately, the miRNAs-TFs-target regulatory network was constructed utilizing the Cytoscape software.

### Survival analysis

GSE29016 gene expression profiling data including 20 SCLC samples and clinical data were obtained from the GEO database (http://www.ncbi.nlm.nih.gov/geo/). The common samples between the SCLC samples and clinical data were screened and removed the samples with survival time less than 1 month. Here, a total of 14 samples were enrolled in the present study.

The sample informations were filtered, and the samples were deleted if the survival time was < 1 month. The samples corresponding to DEGs were screened. The survival package (ver. 2.41-3) in R language and median grouping method were used to conduct the survival analysis. Finally, DEGs under p value < 0.05 were selected to generate the survival curve.

## Results

### Identification of DEGs

**Fig. 1:**
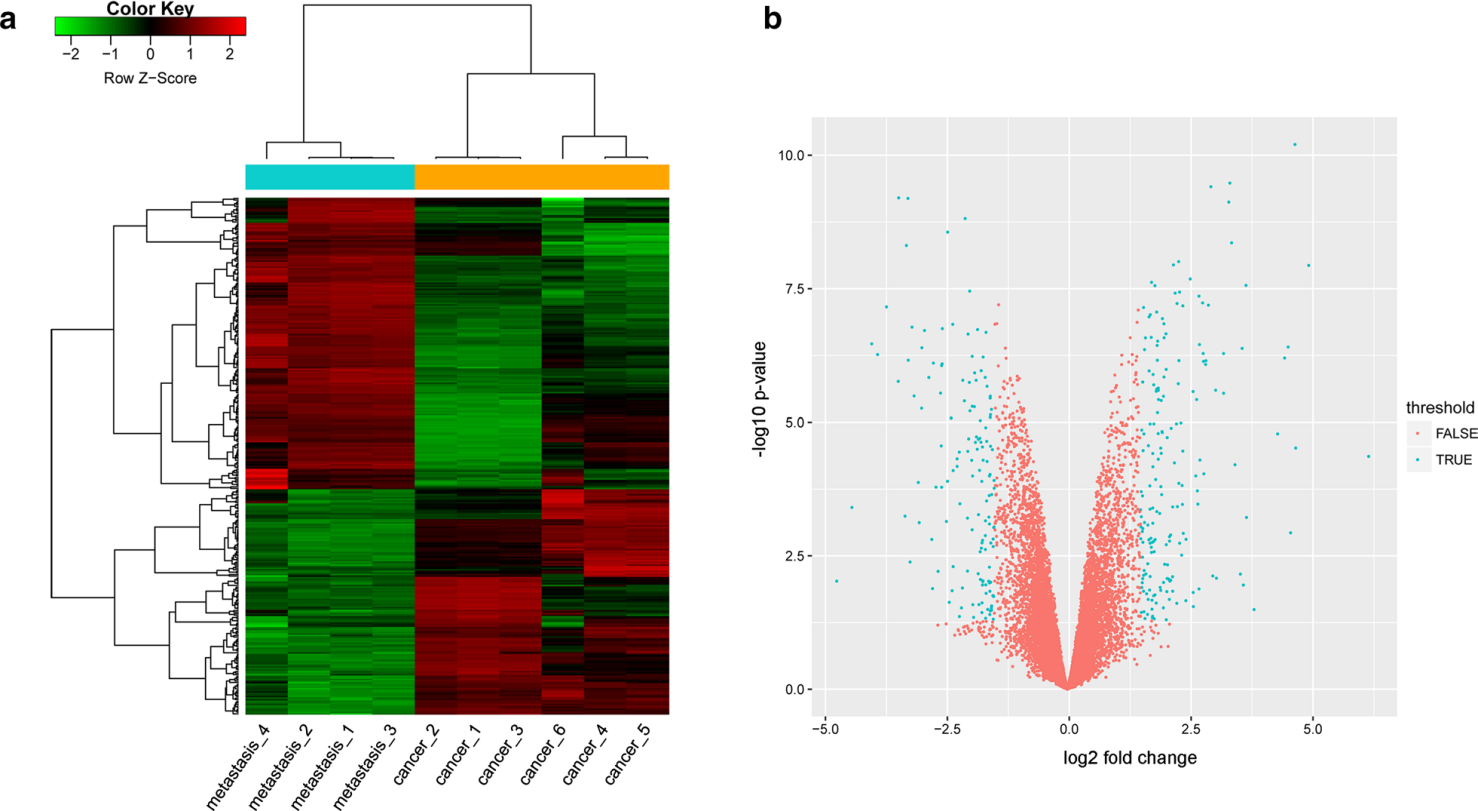
The heatmap and volcano plot of DEGs. **a** The heatmap of DEGs. Green colour corresponds to lowest and red to the highest level of gene expression. By unsupervised clustering, the samples all correctly segregate themselves. **b** Volcano plots for all the genes. The green dots indicate that up- and down-regulated DEGs were significant at p values less than 0.05

We obtained 330 DEGs between the metastasis and cancer groups, of these, 186 were upregulated and 144 were downregulated. The volcano map and heatmap of DEGs were presented in Fig. 1. Here, our results presented that the expressions of MET proto-oncogene, receptor tyrosine kinase (MET), glutamate metabotropic receptor 8 (GRM8), cholinergic receptor nicotinic alpha 5 subunit (CHRNA5), and dachshund family transcription factor 1 (DACH1) were reduced in the SCLC patients with lymph node metastasis compared with those patients without lymph node metastasis. Besides, glutathione-disulfide reductase (GSR), human leukocyte antigen complex P5 (HCP5), and achaete-scute family bHLH transcription factor 1 (ASCL1) were upregulated in the SCLC patients with lymph node metastasis.

### Functional and pathways enrichment analyses

The enriched functions for upregulated DEGs were listed in Table 1a, such as kidney development (GO_BP; p value = 1.89 × 10^−4^), extracellular region (GO_CC; p value = 1.72 × 10^−4^), and calcium ion binding (GO_MF; p value = 2.70 × 10^−4^). The enriched functions for downregulated DEGs were presented in Table 1b, such as nervous system development (GO_BP; p value = 2.57 × 10^−6^), integral component of plasma membrane (GO_CC; p value = 4.14 × 10^−4^), and calcium ion binding (GO_MF; p value = 6.10 × 10^−3^). Here, the upregulated DEGs were not enriched in any pathway. However, downregulated DEGs were attracted to 5 pathways (Table 1c), such as transcriptional misregulation in cancer (pathway; p value = 1.64 × 10^−3^), axon guidance (pathway; p value = 4.07 × 10^−3^), and alcoholism (pathway; p value = 1.29 × 10^−2^). Moreover, a total 6 DEGs were attracted to the pathway of transcriptional misregulation in cancer, such as MET.

**Table 1:** Enrichment analyses for DEGs

Category	Term	Description	P value	Genes
(a) GO functional analysis for upregulated DEGs				
BP	GO:0001822	Kidney development	1.89 × 10^− 4^	SULF1, ITGA8, etc
BP	GO:0043066	Negative regulation of apoptotic process	4.92 × 10^−3^	GCLC, CD38, etc
BP	GO:0045779	Negative regulation of bone resorption	5.80 × 10^−3^	CALCA, CD38, etc
BP	GO:0000302	Response to reactive oxygen species	6.35 × 10^−3^	GPX2, GSR, etc
BP	GO:0038083	Peptidyl-tyrosine autophosphorylation	6.82 × 10^−3^	FRK, LYN, etc
BP	GO:0009725	Response to hormone	7.81 × 10^−3^	GCLC, LYN, etc
BP	GO:0045892	Negative regulation of transcription, DNA-templated	9.56 × 10^−3^	CD38, GCLC, etc
BP	GO:0007169	Transmembrane receptor protein tyrosine kinase signaling pathway	1.43 × 10^−2^	FRK, LYN, etc
BP	GO:0002250	Adaptive immune response	1.48 × 10^−2^	LYN, LAX1, etc
BP	GO:0050853	B cell receptor signaling pathway	1.55 × 10^−2^	CD38, LYN, etc
CC	GO:0005576	Extracellular region	1.72 × 10^−4^	CER1, C3, etc
CC	GO:0005615	Extracellular space	5.81 × 10^−3^	CER1, SELP, etc
CC	GO:0048471	Perinuclear region of cytoplasm	7.73 × 10^−3^	SYT4, LYN, etc
CC	GO:0005886	Plasma membrane	9.00 × 10^−3^	SYT4, CDCP1, etc
CC	GO:0070062	Extracellular exosome	1.21 × 10^−2^	FRK, TSPAN1, etc
CC	GO:0031234	Extrinsic component of cytoplasmic side of plasma membrane	2.85 × 10^−2^	FRK, LYN, etc.
CC	GO:0016942	Insulin-like growth factor binding protein complex	2.89 × 10^−2^	IGF1, IGFBP5
CC	GO:0042567	Insulin-like growth factor ternary complex	3.83 × 10^−2^	IGF1, IGFBP5
CC	GO:0005604	Basement membrane	4.16 × 10^−2^	MATN2, CCDC80, etc
CC	GO:0005578	Proteinaceous extracellular matrix	4.69 × 10^−2^	P3H1, OGN, etc
MF	GO:0005509	Calcium ion binding	2.70 × 10^−4^	ME3, SYT4, etc
MF	GO:0008201	Heparin binding	1.08 × 10^−3^	OGN, SELP, etc.
MF	GO:0032403	Protein complex binding	4.45 × 10^−3^	CALCA, FYB, etc
MF	GO:0004715	Non-membrane spanning protein tyrosine kinase activity	1.06 × 10^−2^	FRK, LYN, etc
MF	GO:0033040	Sour taste receptor activity	1.97 × 10^−2^	PKD2L1, PKD1L3
MF	GO:0004222	Metalloendopeptidase activity	2.59 × 10^−2^	ADAM28, MME, etc
MF	GO:0016668	Oxidoreductase activity, acting on a sulfur group of donors, NAD (P) as acceptor	3.90 × 10^−2^	GSR, TXNRD1
MF	GO:0043208	Glycosphingolipid binding	3.90 × 10^−2^	SELP, LYN
MF	GO:0008237	Metallopeptidase activity	4.63 × 10^−2^	ADAM28, MME, etc.
MF	GO:0000988	Transcription factor activity, protein binding	4.85 × 10^−2^	HEY1, SMAD3
(b) GO analysis for downregulated DEGs				
BP	GO:0007399	Nervous system development	2.57 × 10^−6^	ZC4H2, PCDHB6, etc
BP	GO:0007156	Homophilic cell adhesion via plasma membrane adhesion molecules	4.09 × 10^−6^	CDH7, FAT1, etc
BP	GO:0007268	Chemical synaptic transmission	1.12 × 10^−4^	CBLN1, PCDHB6, etc
BP	GO:0007155	Cell adhesion	2.53 × 10^−4^	EFNB2, SPOCK1, etc
BP	GO:0001764	Neuron migration	1.39 × 10^−3^	ASTN1, RELN, etc
BP	GO:0007411	Axon guidance	8.18 × 10^−3^	NEO1, CDH4, etc
BP	GO:0007416	Synapse assembly	1.20 × 10^−2^	ADGRL3, PCDHB10, etc
BP	GO:0051965	Positive regulation of synapse assembly	1.26 × 10^−2^	LRRN3, LRRN1,
BP	GO:0016339	Calcium-dependent cell–cell adhesion via plasma membrane cell adhesion molecules	2.00 × 10^−2^	PCDHB6, PCDHB11, etc
BP	GO:0045666	Positive regulation of neuron differentiation	2.31 × 10^−2^	SOX11, MMD, etc
CC	GO:0005887	Integral component of plasma membrane	4.14 × 10^−4^	GRIK2, MET, etc
CC	GO:0045211	Postsynaptic membrane	4.08 × 10^−3^	CBLN1, ZC4H2, etc
CC	GO:0005886	Plasma membrane	1.17 × 10^−2^	GRIK2, FHL1, etc
CC	GO:0031941	Filamentous actin	2.05 × 10^−2^	MYO6, FSCN1, etc
CC	GO:0030424	Axon	2.15 × 10^−2^	STMN2, CNR1, etc
CC	GO:0030425	Dendrite	3.31 × 10^−2^	RELN, GNG3, etc
CC	GO:0000788	Nuclear nucleosome	3.93 × 10^−2^	HIST1H2BB, HIST1H3C, etc
CC	GO:0043204	Perikaryon	4.02 × 10^−2^	ASTN1, KCNK1, etc
CC	GO:0034705	Potassium channel complex	4.21 × 10^−2^	KCNA6, KCNK1
CC	GO:0030054	Cell junction	4.64 × 10^−2^	ZC4H2, GRIK2, etc
MF	GO:0005509	Calcium ion binding	6.10 × 10^−3^	CDH7, DGKB, etc
MF	GO:0032051	Clathrin light chain binding	3.60 × 10^−2^	NSG1, HMP19
MF	GO:0009931	Calcium-dependent protein serine/threonine kinase activity	5.00 × 10^−2^	CAMK4, DCX

Term	Description	Count	P value	Key genes
(c) KEGG pathway analysis for downregulated DEGs				
hsa05202	Transcriptional misregulation in cancer	6	1.64 × 10^−3^	HIST1H3J, EYA1, MET, ETV1, HIST1H3C, MEIS1
hsa04360	Axon guidance	5	4.07 × 10^−3^	EPHA4, PAK3, EFNB2, MET, DPYSL5
hsa05034	Alcoholism	5	1.29 × 10^−2^	HIST1H3J, HIST1H2BB, CAMK4, HIST1H3C, GNG3
hsa04723	Retrograde endocannabinoid signaling	4	1.53 × 10^−2^	SLC32A1, GABRG2, CNR1, GNG3
hsa05322	Systemic lupus erythematosus	4	3.20 × 10^−2^	HIST1H3J, HIST1H2BB, HIST1H3C, HLA-DQA1

DEGs, differentially expressed genes; GO, Gene Ontology; BP, biological process; CC, cellular component; MF, molecular function; KEGG, Kyoto Encyclopedia of Genes and Genomes

### PPI network and module analyses

**Fig. 2:**
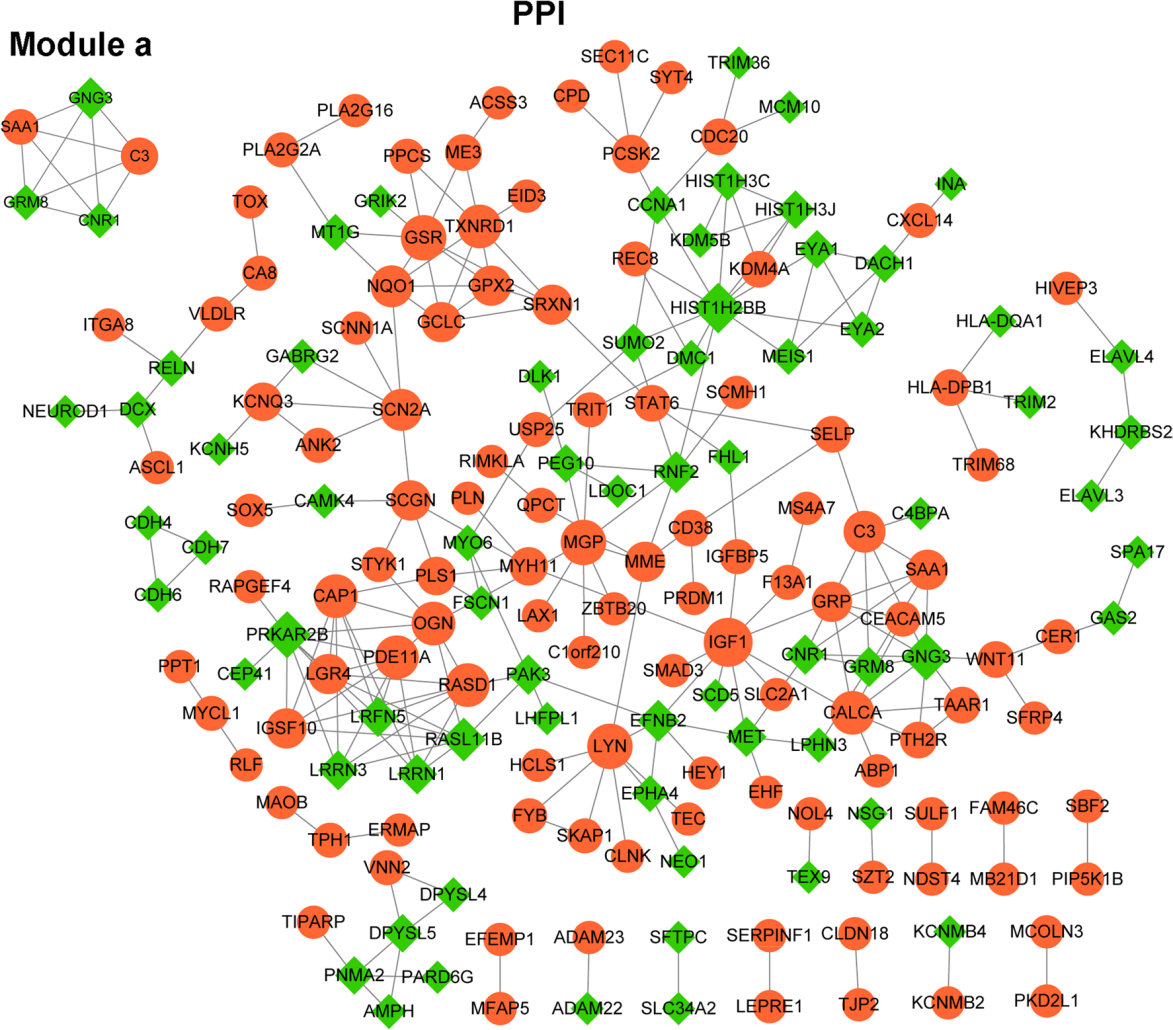
PPI network and subnetwork module of the DEGs. The orange roundness stands for the upregulated DEGs and the green rhombus stands for the downregulated DEGs. PPI, protein–protein interaction; DEGs, differentially expressed genes

There were 178 nodes and 237 interaction pairs in the PPI network (Fig. 2). Afterwards, one subnetwork module a (score = 5) with 5 nodes and 10 interaction pairs was obtained through the MCODE (score ≥ 5) plugin in Cytoscape software. According to the degree of DEGs in the PPI network, the top 10 DEGs were selected, then the GO-BP analysis for the top 10 DEGs and module a DEGs were implemented. The top 10 DEGs in the PPI network and DEGs in the module a are presented in Table 2. The enriched functions for the DEGs in the PPI network were shown in Table 3, such as homeostatic process (GO_BP; p value = 5.44 × 10^−5^), regulation of phosphorylation (GO_BP; p value = 1.53 × 10^−4^), and regulation of phosphate metabolic process (GO_BP; p value = 1.78 × 10^−4^). Meanwhile, the enriched functions for module a DEGs were listed in Table 3, such as G-protein coupled receptor protein signaling pathway (GO_BP; p value = 2.14 × 10^−3^), cell surface receptor linked signal transduction (GO_BP; p value = 9.26 × 10^−3^), and regulation of inflammatory response (GO_BP; p value = 2.23 × 10^−2^). Here, our results showed that GRM8 was attracted to the G-protein coupled receptor signaling pathway.

**Table 2:** The top 10 DEGs in the PPI network and module a

Gene	Degree	Regulate
PPI		
HIST1H2BB	11	Down
IGF1	10	Up
PRKAR2B	9	Down
GNG3	9	Down
GSR	8	Up
LYN	8	Up
MGP	8	Up
CALCA	7	Up
TXNRD1	7	Up
Module a		
GNG3	9	Down
C3	6	Up
GRM8	5	Down
SAA1	5	Up
CNR1	5	Down

DEGs, differentially expressed genes; PPI, protein–protein interaction

**Table 3:** Enrichment analyses for DEGs in the PPI network and subnetwork module a

Term	Description	Count	P value	Key genes
PPI				
GO:0042592	Homeostatic process	6	5.44 × 10^−5^	CALCA, GSR, LYN, IGF1, TXNRD1, GNG3
GO:0042325	Regulation of phosphorylation	5	1.53 × 10^−4^	CALCA, PRKAR2B, LYN, IGF1, GNG3
GO:0019220	Regulation of phosphate metabolic process	5	1.78 × 10^−4^	CALCA, PRKAR2B, LYN, IGF1, GNG3
GO:0051174	Regulation of phosphorus metabolic process	5	1.78 × 10^−4^	CALCA, PRKAR2B, LYN, IGF1, GNG3
GO:0009725	Response to hormone stimulus	4	1.47 × 10^−3^	PRKAR2B, LYN, MGP, GNG3
GO:0009719	Response to endogenous stimulus	4	1.96 × 10^−3^	PRKAR2B, LYN, MGP, GNG3
GO:0019725	Cellular homeostasis	4	2.92 × 10^−3^	CALCA, GSR, TXNRD1, GNG3
GO:0043085	Positive regulation of catalytic activity	4	3.99 × 10^−3^	CALCA, PRKAR2B, GNG3, CAP1
GO:0001932	Regulation of protein amino acid phosphorylation	3	5.52 × 10^−3^	PRKAR2B, LYN, IGF1
GO:0044093	Positive regulation of molecular function	4	5.58 × 10^−3^	CALCA, PRKAR2B, GNG3, CAP1
Module a				
GO:0007186	G-protein coupled receptor protein signaling pathway	4	2.14 × 10^−3^	C3, GRM8, CNR1, GNG3
GO:0007166	Cell surface receptor linked signal transduction	4	9.26 × 10^−3^	C3, GRM8, CNR1, GNG3
GO:0050727	Regulation of inflammatory response	2	2.23 × 10^−2^	C3, SAA1
GO:0002526	Acute inflammatory response	2	2.87 × 10^−2^	C3, SAA1
GO:0007204	Elevation of cytosolic calcium ion concentration	2	3.21 × 10^−2^	SAA1, GNG3
GO:0051480	Cytosolic calcium ion homeostasis	2	3.44 × 10^−2^	SAA1, GNG3
GO:0032101	Regulation of response to external stimulus	2	4.62 × 10^−2^	C3, SAA1

DEGs, differentially expressed genes; PPI, protein–protein interaction

### MiRNAs-TFs-target regulatory network analyses

**Fig. 3:**
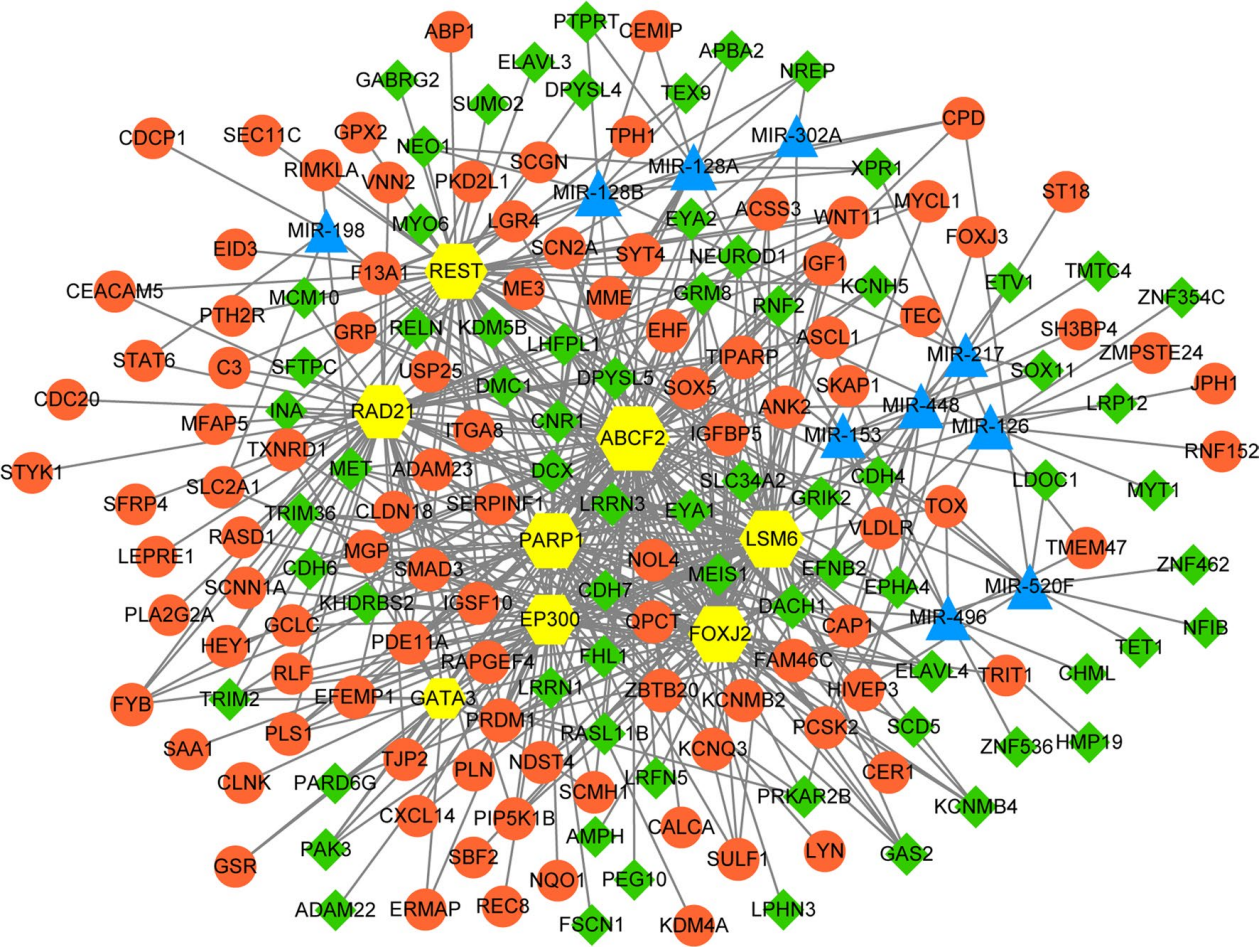
The miRNAs-TFs -target regulatory network. The orange roundness presents the upregulated DEGs, and the green rhombus presents the downregulated DEGs. The light blue triangle stands for miRNAs, and the yellow hexagon stands for TFs. miRNA, microRNA; TFs, transcription factors; DEGs, differentially expressed genes

The miRNAs-TFs-target regulatory network was established with 8 TFs, 10 miRNAs and 187 DEGs through the Cytoscape software (Fig. 3). MiR-126 was identified in the miRNAs-TFs-target regulatory network. A total of 11 genes were regulated by miR-126 in our study, such as GRM8 and DACH1 (Table 4).

**Table 4:** Genes associated with miR-126 in miRNAs-TFs-target regulatory network

miRNAs	Genes
miR-126	ZMPSTE24
miR-126	DACH1
miR-126	EYA1
miR-126	RNF152
miR-126	GRM8
miR-126	ZNF354C
miR-126	MYT1
miR-126	PCSK2
miR-126	JPH1
miR-126	TMEM47
miR-126	XPR1

### Survival analysis

**Fig. 4:**
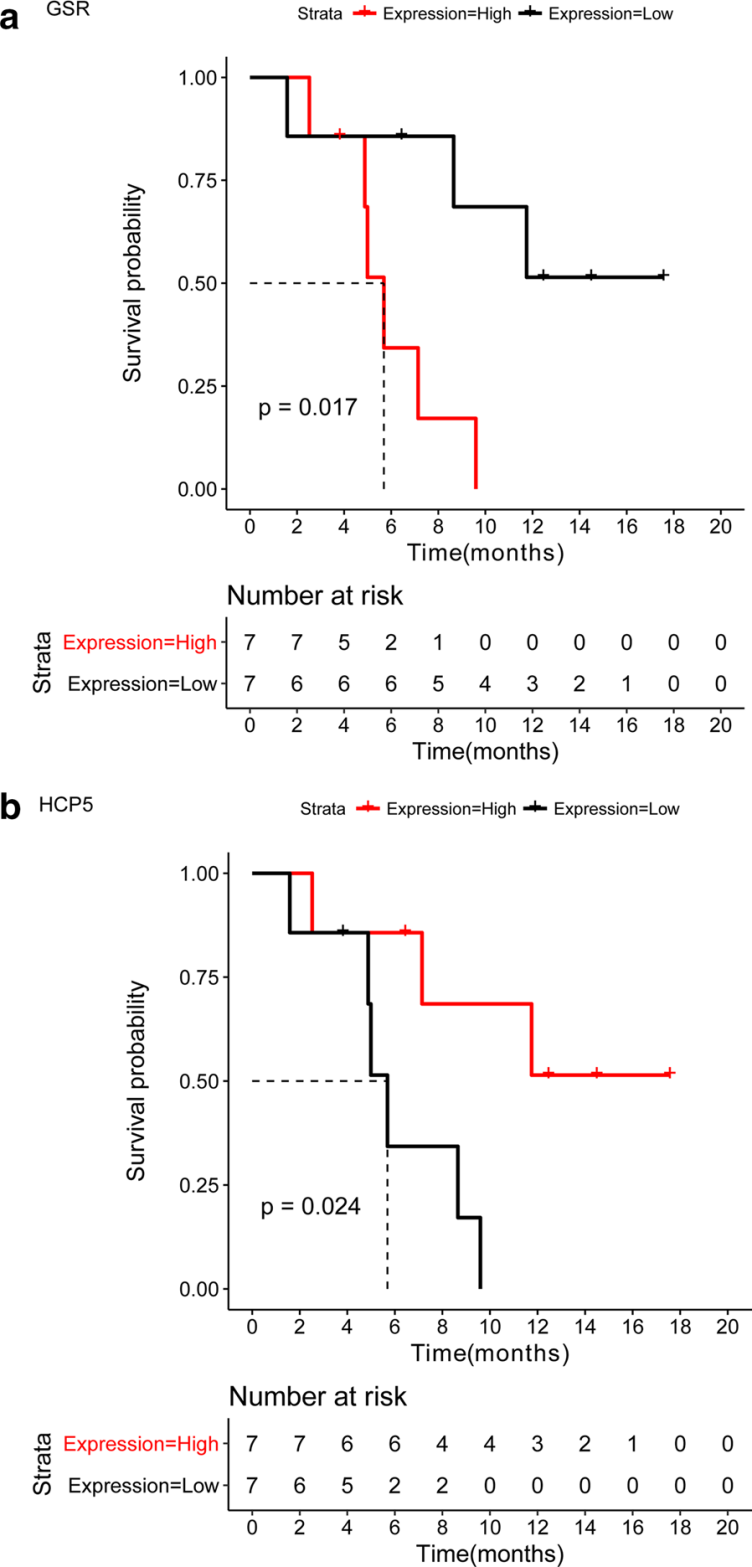
The survival curve for the GSR (**a**) and HCP5 (**b**). The horizontal axis represents the survival time (months), and the vertical axis presents the survival rate. The red curve stands for the group of upregulated gene expression (high expression), and the black curve represents the group of downregulated gene expression (low expression). A p value of < 0.05 was condidered statistically significant between upregulated and downregulated gene expression. GSR, glutathione-disulfide reductase; HCP5, human leukocyte antigen complex P5

Total 164 DEGs have the corresponding sample information. Hence, the 164 DEGs were used for generating the survival curve. There were 2 DEGs correlated with the survival of SCLC, such as GSR and HCP5 (Fig. 4).

## Discussion

SCLC is a highly malignant cancer, and over 70% of patients with SCLC present with the metastatic disease [5]. But the molecular determinants of SCLC metastasis are unknown. In the current study, some novel DEGs and miRNA associated with the lymph node metastasis of SCLC were obtained via the comprehensive bioinformatical analyses, such as miR-126, DACH1, GRM8, MET, RSD and HCP5.

In SCLC, more than 95% of patients have a smoking history and their 5-year survival rates are under 2% [29]. As known, a variety of addictive compound nicotine was contained in tobacco, which begins with the binding of nicotine to the nicotinic acetylcholine receptor (nAChR). In especial, the nAChR gene cluster encoding the α3, α5 and β4 nAChR subunits such as CHRNA5/A3/B4 was differentially expressed in SCLC [30]. In addition, ASCL1 which is a transcription factor implicated in the pathogenesis of SCLC is overexpressed in SCLC [31]. Similarly, CHRNA5 and ASCL1 were differentially expressed in SCLC with the metastatic disease. Interestingly, ASCL1 might regulate the expression of the clustered nAChR genes. Therefore, positive control genes have validated that the analysis pipeline is doable.

In general, lymph node metastasis of LC is positively related to lymphangiogenesis [32]. Currently, there is no direct proofs present that miR-126 is significantly associated with the lymph node metastasis of SCLC. However, Sasahira et al. found that the downregulated miR-126 was correlated with the induction of lymphangiogenesis in the OSCC [33]. It has been uncovered that miR-126 is downregulated in primary SCLC tumor samples [34]. In addition, miR-126 has an negative effect on the growth and proliferation of SCLC cells [35]. Meanwhile, miR-126 is also a crucial regulator for the vessel development [36]. Here, miR-126 was identified in the miRNAs-TFs-target regulatory network. These findings all indicated that miR-126 is likely to participate in the lymph node metastasis process of SCLC through inducing the lymphangiogenesis.

A total of 11 genes were regulated by miR-126 in our study, such as GRM8 and DACH1. DACH1, implicated in the suppression of tumor growth, is downregulated in human malignancies, such as LC [37]. It is reported that the LC invasion and tumor growth can be inhibited by DACH1 through suppressing the CXCL5 signaling [38]. Here, our results presented that DACH1 expression was downregulated in the SCLC patients with lymph node metastasis. Probably, DACH1 decrease inhibits the lymph node metastasis process of SCLC. GRM8, encoded by the GRM8 gene, are a family of G protein-coupled receptors. Here, our results showed that GRM8 was downregulated in the SCLC patients with lymph node metastasis and was attracted to the G-protein coupled receptor signaling pathway. However, the role of GRM8 in the lymph node metastasis process of SCLC remains unclear. Previous studies indicated that signaling pathways controlled by GPCRs facilitate proliferation, cell migration, angiogenesis, and inflammation [39]. Namely, GPCRs are likely to promote the migration of LC cells into the lymph node. Therefore, we speculated that GRM8 was likely to participate in the lymph node metastasis process of SCLC.

The gene expression programs are controlled by a variety of TFs, and its misregulation result in various diseases [40]. Briefly, the transcriptional misregulation can cause thousands of diseases. Here, a total of 6 DEGs were attracted to the pathway of transcriptional misregulation in cancer, such as MET. It has been revealed that MET regulates the remodeling and morphogenesis of tissues, and its dysregulation is implied in the oncogenic signaling and metastasis [41, 42]. Here, MET was regulated in the SCLC patients with lymph node metastasis. Thus, MET dysregulation may participate in the lymph node metastasis process of SCLC through the transcriptional misregulation in cancer pathway.

GSR encoded a member of the class-I pyridine nucleotide-disulfide oxidoreductase family, which played a key role in cellular antioxidant defense. GSR reduced oxidized glutathione disulfide (GSSG) to the sulfhydryl form glutathione (GSH). Whereas, GSH played complex roles in cancer, including the protective and pathogenic roles [43]. In a previous study, GSR expression level was significantly increased in tumor tissue from patients with LC [44]. Although there was no direct proofs to indicate GSR association with the lymph node metastasis process of SCLC, the glioblastoma multiforme patients with high GSR expression showed poor survival [45]. Similarly, GSR was up-regulated in the patients with lymph node metastasis process of SCLC and displayed a poor survival results. Therefore, we speculated that GSR might have a central role in the lymph node metastasis process of SCLC.

In addition, HCP5 was significantly down-regulated in patients with ovarian cancer [46]. Similarly, HCP5 was also down-expressed in patients with lung adenocarcinoma. However, Teng et al. reported that HCP5 was up-regulated in glioma tissues as well as in U87 and U251 cells [47]. In addition, they found that HCP5 regulated the glioma cells malignant proliferation through binding to miR-139 by up-regulating RUNX1. Probably, HCP5 expression was different in various cancers. In the present study, HCP5 was upregulated in the lymph node metastasis process of SCLC. Hence, we speculated that GSR and HCP5 may involve in the lymph node metastasis process of SCLC. However, the predicted results cannot be verified by laboratory data due to the limitation of sample extraction. In further studies, we will confirm the expressions of the above discussed DEGs and miRNA once we collected the sufficient samples.

## Conclusion

In summary, our results suggested that miR-126 and its target gene DACH1 may implicate in the lymph node metastasis process of SCLC. Additionally, GRM8, MET, RSD and HCP5 were implicated in the lymph node metastasis process of SCLC.

## Abbreviations

SCLC: small cell lung cancer
DEGs: differentially expressed genes
LC: lung cancer
KEGG: Kyoto Encyclopedia of Genes and Genomes
PPI: protein–protein interaction
STRING: Search Tool for the Retrieval of Interacting Genes
GRM8: glutamate metabotropic receptor 8
DACH1: dachshund family transcription factor 1
GSR: glutathione-disulfide reductase
HCP5: human leukocyte antigen complex P5
